# KCC2 Chloride Transport Contributes to the Termination of Ictal Epileptiform Activity

**DOI:** 10.1523/ENEURO.0208-20.2020

**Published:** 2021-03-08

**Authors:** Volodymyr I. Dzhala, Kevin J. Staley

**Affiliations:** 1 Department of Neurology, Massachusetts General Hospital, Boston, MA 02114; 2 Harvard Medical School, Boston, MA 02114

**Keywords:** chloride, CLP257, GABA, KCC2, seizure, VU0463271

## Abstract

Recurrent seizures intensely activate GABA_A_ receptors (GABA_A_-Rs), which induces transient neuronal chloride ([Cl^–^]_i_) elevations and depolarizing GABA responses that contribute to the failure of inhibition that engenders further seizures and anticonvulsant resistance. The K^+^-Cl^–^ cotransporter KCC2 is responsible for Cl^–^ extrusion and restoration of [Cl^–^]_i_ equilibrium (E_Cl_) after synaptic activity, but at the cost of increased extracellular potassium which may retard K^+^-Cl^–^ extrusion, depolarize neurons, and potentiate seizures. Thus, KCC2 may either diminish or facilitate seizure activity, and both proconvulsant and anticonvulsant effects of KCC2 inhibition have been reported. It is now necessary to identify the loci of these divergent responses by assaying both the electrographic effects and the ionic effects of KCC2 manipulation. We therefore determined the net effects of KCC2 transport activity on cytoplasmic chloride elevation and Cl^–^ extrusion rates during spontaneous recurrent ictal-like epileptiform discharges (ILDs) in organotypic hippocampal slices *in vitro*, as well as the correlation between ionic and electrographic effects. We found that the KCC2 antagonist VU0463271 reduced Cl^–^ extrusion rates, increased ictal [Cl^–^]_i_ elevation, increased ILD duration, and induced status epilepticus (SE). In contrast, the putative KCC2 upregulator CLP257 improved chloride homeostasis and reduced the duration and frequency of ILDs in a concentration-dependent manner. Our results demonstrate that measuring both the ionic and electrographic effects of KCC2 transport clarify the impact of KCC2 modulation in specific models of epileptiform activity. Anticonvulsant effects predominate when KCC2-mediated chloride transport rather than potassium buffering is the rate-limiting step in restoring E_Cl_ and the efficacy of GABAergic inhibition during recurrent ILDs.

## Visual Abstract

**Figure:**
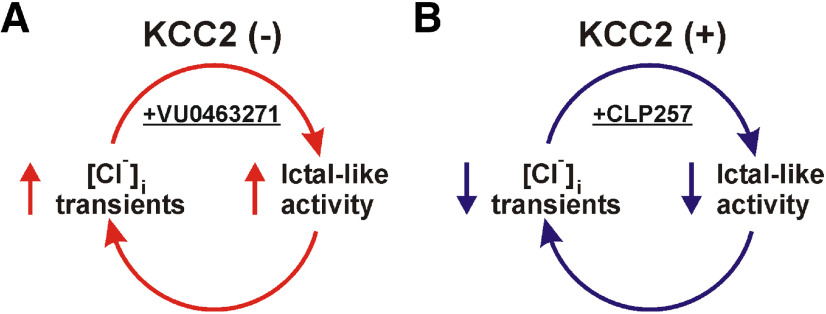

## Significance Statement

In an *in vitro* preparation that generates spontaneous ictal-like epileptiform discharges (ILDs), we measured the effects of acute KCC2 modulation on both neuronal chloride and ILD activity. We demonstrate that inhibiting KCC2 enhances ictal elevations in [Cl^–^]_i_, reduces extrusion and prolongs ILDs. Enhancing KCC2 activity reduces ictal [Cl^–^]_i_ elevations and ILD duration. These findings regarding the role of KCC2 on baseline chloride, chloride elevations during ILD activity, Cl^–^ extrusion rates and ILD activity resolve conflicting reports in the literature, provide a coherent understanding of the role KCC2 activity in chloride homeostasis during ILDs, and support the feasibility of developing KCC2 modulators into sorely-needed anticonvulsant medications.

## Introduction

Recurrent seizures often respond poorly to first line medications that target the inhibitory chloride-permeable GABA_A_ receptor (GABA_A_-R; Painter et al., 1999; Fung et al., 2019). Low intracellular chloride concentration ([Cl^–^]_i_) is an important determinant of inhibitory postsynaptic GABA_A_-R signaling. This low [Cl^–^]_i_ is established by a Gibbs–Donnan system comprised of impermeant cytoplasmic and extracellular anions, and a membrane permeability for chloride salts and water provided by equilibrative cation-chloride cotransporters (CCCs; Delpire and Staley, 2014; Glykys et al., 2014). This type of system can be characterized by the reversal potential for membrane chloride currents (E_Cl_) when the system is at equilibrium. GABA_A_-R-gated chloride currents alter the local [Cl^–^]_i_ and move this system away from equilibrium. Restoration of equilibrium after synaptic signaling is achieved by the flux of chloride salts through the CCCs, such as the K^+^-Cl^–^ cotransporter KCC2 (transporting 1 K^+^ and 1 Cl^–^ ion per cycle) and Na^+^-K^+^-2Cl^–^ cotransporter 1 (NKCC1; transporting 1 Na^+^, 1 K^+^, and 2 Cl^–^ ions per cycle; Gamba, 2005). Because GABA_A_-gated chloride currents are “downhill,” that is, the chloride diffuses along its electrochemical gradient, the restoration of the baseline [Cl^–^]_i_ requires energy; this is applied via cation cotransport. For KCC2, forward transport (canonical Cl^–^ extrusion) is driven by downhill potassium extrusion that increases extracellular potassium ([K^+^]_0_). For NKCC1, forward transport (canonical Cl^–^ import) is driven by downhill sodium import that increases intracellular sodium. The restoration of K^+^ and Na^+^ gradients ultimately requires energy in the form of ATP as Na^+^-K^+^-ATPase-mediated cation transport that restores the Na^+^ and K^+^ gradients.

High rates of synaptic Cl^–^ influx, as occurs during seizures, stresses neuronal Cl^–^ homeostasis (Staley and Proctor, 1999). To restore [Cl^–^]_i_ to baseline, high rates of CCC-mediated Cl^–^ efflux are required. But at these high cotransport rates, K^+^ efflux is also increased, which may elevate [K^+^]_0_ (Viitanen et al., 2010). This may change the equilibrium conditions for CCC transport, leading to higher [Cl^–^]_i_. In turn, this may lead to proconvulsant depolarizing GABA conductance, while the elevated [K^+^]_0_ may depolarize neurons directly, predisposing to further seizures (González et al., 2015). Given these secondary effects of CCCs, it may not be surprising that there are conflicting results regarding the role of CCCs in seizures. When *in vitro* epileptiform activity was induced with 4-AP, blocking KCC2 transport activity with either VU0240551 or high doses of bumetanide abolished ictal-like epileptiform discharges (ILDs), while enhancing KCC2 activity with a high concentration of CLP257 increased the duration of ILDs (Hamidi and Avoli, 2015). On the other hand, there are both genetic and pharmacological data supporting an anticonvulsant role of KCC2 expression in chronic temporal lobe epilepsy. For example, selective inhibition of KCC2 with VU0463271 led to hyperexcitability and epileptiform discharges in hippocampal slices *in vitro* exposed to low magnesium, and induced seizures *in vivo* (Sivakumaran et al., 2015). Human loss of function mutations of KCC2 result in febrile seizures or more severe early infantile epileptic encephalopathies (Kahle et al., 2016; Duy et al., 2019). Experimental studies of gain of function mutations in KCC2 have reported resistance to 4-AP induced ILDs, lower baseline [Cl^–^]_i_, and higher Cl^–^ extrusion rates after exposure to glutamate (Moore et al., 2018). However, since glutamate massively increases [K^+^]_0_ (Vargova et al., 2001) creating a proportionate increase in equilibrium [Cl^–^]_i_, increased KCC2 activity would only alter Cl^–^ extrusion in recorded cells that were not in areas of high [K^+^]_0_; how this relates to ictal conditions (Heinemann et al., 1977) is therefore unclear (Lux and Heinemann, 1978).

Here, we studied KCC2 transport activity during recurrent ILDs in an *in vitro* model of epileptogenesis that does not require exogenous convulsant conditions (Berdichevsky et al., 2012) and in which both [Cl^–^]_i_ and electrographic epileptiform activity could be monitored. We describe the effects of ILDs on [Cl^–^]_i_. We measured the effects of the low and high affinity KCC2 inhibitors furosemide and VU463271 (Delpire et al., 2012), as well as the putative KCC2 activator CLP257 (Gagnon et al., 2013; Cardarelli et al., 2017) on epileptiform activity as well as neuronal chloride elevation and extrusion rates during spontaneous ILDs.

## Materials and Methods

All animal-use protocols were in accordance with the guidelines of the National Institutes of Health and the Massachusetts General Hospital Center for Comparative Medicine on the use of laboratory animals. All protocols were approved by the Subcommittee on Research and Animal Care (SRAC).

### Culture of organotypic hippocampal slices and experimental conditions

Transverse 350-μm hippocampal slices were prepared from C57BL/6 and CLM1 (Duke University Medical Center, Durham, NC) mice at postnatal day (P)6–P7 as previously described (Dyhrfjeld-Johnsen et al., 2010; Berdichevsky et al., 2012). Acute slices were mounted on poly-l-Lysine coated glass coverslips (Electron Microscopy Sciences). Slices were incubated in 1000 μl of NeuroBasal/B27(1×) medium (Invitrogen by Life Technologies) supplemented with 0.5 mm GlutaMAX and 30 μg/ml gentamicin (all from Invitrogen) in six-well plates with low-evaporation lid (Becton Dickinson Labware), in a humidified 37°C atmosphere that contained 5% CO_2_, placed on a rocking platform (less than one cycle per minute). Culture medium was changed bi-weekly. For acute recordings and imaging, slices were transferred to a submerged chamber and continuously superfused in oxygenated (95% O_2_ and 5% CO_2_) artificial CSF (ACSF) containing the following: 126 mm NaCl, 3.5 mm KCl, 2 mm CaCl_2,_ 1.3 mm MgCl_2_, 25 mm NaHCO_3_, 1.2 mm NaHPO_4_, and 11 mm glucose (pH 7.4) at 32 ± 0.5°C and a flow rate of 2 ml/min. All organotypic hippocampal slices were used at days *in vitro* (DIV)1–DIV28.

Pharmacological agents included the Bumetanide at a concentration (200 μm) that blocks NKCC1 and KCC2, the less selective cation-chloride cotransport inhibitor furosemide (0.1 and 1 mm), the specific KCC2 blockers VU0463271 (0.1 and 1 μm) and VU0240551 (10 μm), the putative KCC2 cotransporter activator CLP257 (1 μm), the GABA_A_ receptor antagonist SR95531 (10 μm), and the sodium voltage gated channel antagonist tetrodotoxin (TTX; 1 μm). Bumetanide, furosemide, and SR95531 were from Sigma-Aldrich. VU0240551, VU0463271, CLP257, and TTX were from Tocris Bioscience.

### Electrophysiological recordings and data analysis

Extracellular field potentials were recorded in the CA3 and CA1 pyramidal cell layer of organotypic hippocampal slices using custom-made tungsten-coated 50-μm wire microelectrodes. The electrical signals were digitized using an analog-to-digital converter DigiData 1322A (Molecular Devices, Inc). AxoScope 10.7 and Clampfit 10.7 (Molecular Devices), Origin 2018 (OriginLab Corporation) and SigmaPlot 11.0 (Systat Software, Inc) programs were used for data acquisition and analyses. Recordings were sampled at 10 kHz. Interictal epileptiform discharges (IEDs) were defined as synchronous network-driven bursts characterized by short (0.1–3 s) duration and large amplitude population spikes. The frequency, duration and amplitude of IEDs substantially varied between recurrent ILDs. ILDs were defined as hyper-synchronous, large-amplitude and high-frequency population spikes followed by sustained ictal-tonic and/or intermittent ictal-clonic after- discharges, with the duration of the population spikes and after-discharge complex lasting >5 s. Power spectrum analysis was performed on the electrical recordings after filtering with a Bessel high pass filter of 1 Hz and applying a Hamming window function. The power of the electrical activity was calculated by integrating the root mean square value of the signal amplitude in corresponding time windows and frequency range from 1 to 1000 Hz. For comparison between slices, power was normalized for each slice with the highest value in control conditions.

### Two-photon imaging of Clomeleon, quantitative and morphologic analysis

Neuronal chloride concentration was determined in CA1 pyramidal neurons expressing the ratio-metric chloride indicator Clomeleon (Kuner and Augustine, 2000). High-resolution two-photon excitation laser scanning imaging of the Cl^–^-sensitive yellow fluorescent protein (YFP) and the Cl^–^-insensitive cyan fluorescent protein (CFP) was performed on an Olympus Fluoview 1000 MPE microscope. A mode-locked titanium-sapphire laser (MaiTai, Spectra Physics) with 860-nm two-photon excitation was used to generate fluorescence. Emitted light passed through a dichroic mirror and was bandpass filtered through 480 ± 15 nm (D480/30) for CFP and a 535 ± 20-nm filter (D535/40) for YFP (FV10-MRCYR/XR). Time series acquisition of 720 frames (256 × 256 pixels for 254.46 × 254.46 μm) with 5- to 10-s intervals was performed to measure chloride concentration as a function of time in control conditions, during a 30- to 60-min period of applications of drugs, and over a 30- to 60-min period of wash-out.

For morphologic analysis, organotypic slices were imaged through the CA1 pyramidal cell layer (*z*-axis dimension 0–100 μm, 1- to 2-μm step size). ImageJ 1.51 software (National Institutes of Health) was used for quantitative analysis. Regions of interest (ROIs) were selected using the chloride insensitive CFP fluorescence. The ratio of the YFP/CFP fluorescence intensity was used for [Cl^–^]_i_ calculation (Kuner and Augustine, 2000; Berglund et al., 2008; Glykys et al., 2009). The CFP emission of Clomeleon was used for the high-resolution morphologic analysis (Dzhala et al., 2012).

### Statistical analysis

Group measures are expressed as mean ± SD or median (25−75%) ± SD as indicated. The Shapiro–Wilk test was used to determine normality of the data. The Student’s *t* test (paired or unpaired) was performed for parametric comparison of normally distributed data. The Wilcoxon signed-rank test (paired data) and Mann–Whitney test (unpaired data, two-tail) were used for non-parametric comparison of arbitrary distributed data. One-way repeated measures (RM) ANOVA was used for multiple comparison of parametric data to evaluate the differences in the mean values among the control and treatment groups. The Friedman RM ANOVA on ranks was used for non-parametric data to determine the differences in the median values among the control and treatment groups. The Tukey’s test was used for all pairwise comparisons of the responses to the different treatment groups. The level of significance was set at *p* < 0.05.

## Results

### Chloride transients during ILDs and baseline chloride changes during epileptogenesis

**Figure 1.**
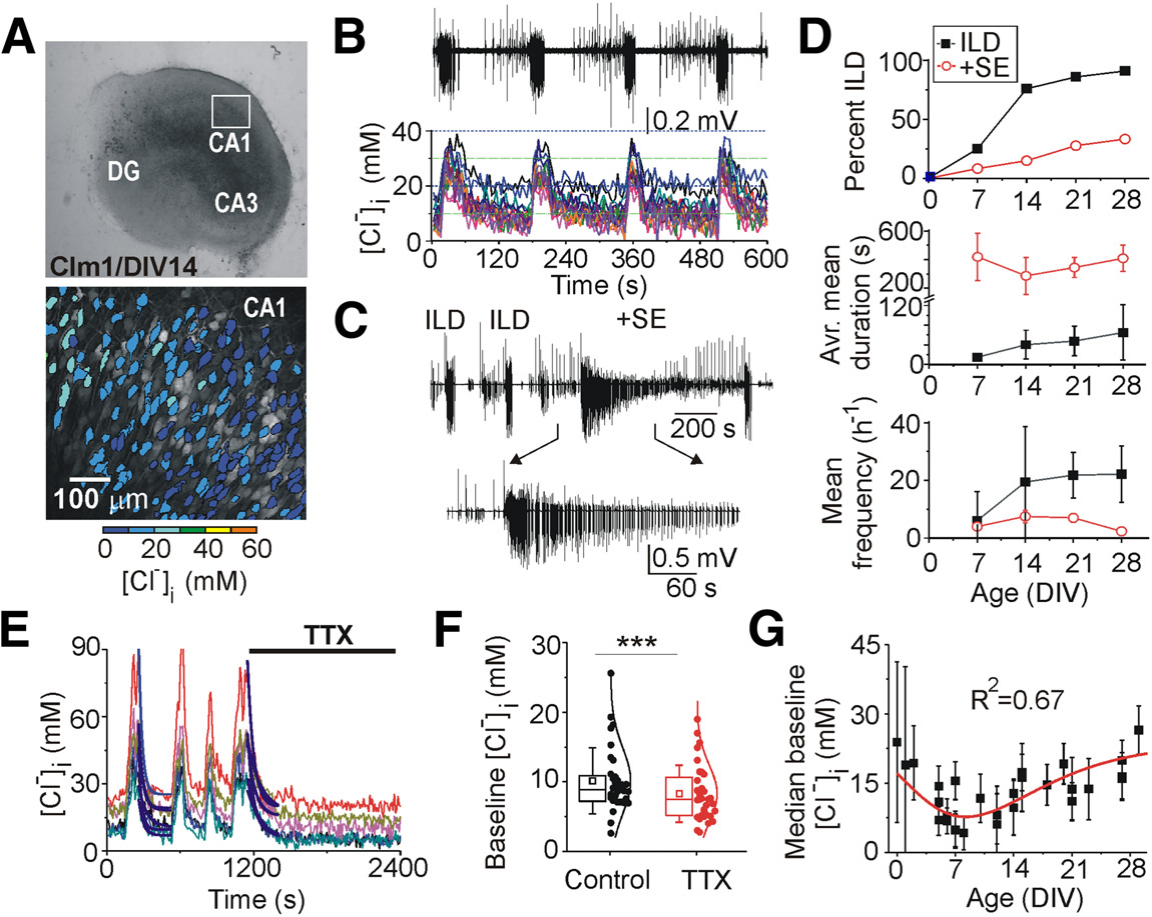
Neuronal chloride accumulation during spontaneous ILDs and epileptogenesis in organotypic hippocampal slices *in vitro*. ***A***, Microphotograph of incubated organotypic hippocampal slice from CLM1 mice captured at DIV14 and corresponding two-photon fluorescence image of the CA1 pyramidal cell layer. Overlays of 50 imaging planes from 0 to 100 μm below the slice surface (2-μm steps) are shown. Neuronal cell bodies are pseudo colored according to [Cl^–^]_i_ (Dzhala et al., 2010, 2012). ***B***, Simultaneous extracellular field potential recording and two-photon fluorescence chloride imaging were used to monitor neuronal network activity and [Cl^–^]_i_ as a function of time. Example of spontaneous recurrent ILDs and corresponding [Cl^–^]_i_ transients in the CA1 pyramidal cells at DIV14. ***C***, Recurrent ILDs can last from 10 to 20 s to >5 min, the ILD duration threshold for SE. Example of recurrent ILDs and SE at DIV14. ***D***, Percentage of slices with spontaneous recurrent ILDs and SE, and corresponding mean duration and frequency of ILDs as a function of age. ***E***, [Cl^–^]_i_ transients in the CA1 pyramidal cells during recurrent ILDs at DIV14. The sodium channel blocker TTX (1 μm) rapidly abolished recurrent ILDs and corresponding [Cl^–^]_i_ transients, and significantly reduced the base line [Cl^–^]_i_. Exponential fits (y = A1exp(-x/t1) + y0) were used to measure base line [Cl^–^]_i_ (y0) in individual cells (solid navy curves) during recovery from ILD (adj. *R*^2^ = 0.9–0.96). ***F***, Box (left) + data (right) plots correspond to median (25−75%) [Cl^–^]_i_ in paired cells (filled circles) and their distribution curves; open squares and whisker range indicate mean ± SD; ***p < 0.001 (paired sample Wilcoxon signed-ranks test). ***G***, Median baseline [Cl^–^]_i_ in individual slices as a function of age (DIV0–DIV28).

Organotypic hippocampal slice cultures from CLM-1 mice expressing the genetically encoded intracellular chloride fluorophore Clomeleon were used as a model of traumatic brain injury and epileptogenesis *in vitro* (Dyhrfjeld-Johnsen et al., 2010; Dzhala et al., 2012; Dzhala and Staley, 2015). Hippocampal slices were incubated for three to four weeks. Non-invasive extracellular field potential recordings and two-photon fluorescence Clomeleon imaging were performed in the CA1 pyramidal cell layer to monitor neuronal network activity and [Cl^–^]_i_ (Fig. 1*A*,*B*). The first week latent period was followed by spontaneous ILDs and status epilepticus (SE). Recurrent ILDs were characterized by an initial spike-and-wave bursts followed by secondary ictal tonic-clonic discharges, and subsequent postictal depression. SE was defined as continuous ILDs for at least 5 min, or by sustained ictal-like tonic-clonic epileptiform discharges without recovery to baseline activity between the ILDs (Fig. 1*C*). The incidence of spontaneous ILDs and SE, and the mean duration and frequency of ILDs progressively increased during epileptogenesis (Fig. 1*D*). The averaged mean duration of ILDs increased from 15 ± 2.6 s at DIV6–DIV8 (*N* = 12 slices) to 64.9 ± 56.2 s at DIV27–DIV28 (*N* = 11 slices), and the mean frequency of these ILDs increased from 6 ± 10.2 ILDs per hour at DIV6–DIV8, 21 ± 9.7 ILDs per hour at DIV27–DIV28 (Fig. 1*D*).

Two-photon imaging of Clomeleon was performed during extracellular field potential recordings (Fig. 1*B*). Under control conditions, baseline [Cl^–^]_i_ distribution in CA1 pyramidal cells varied from 5 to 20 mm (Fig. 1*A*,*B*). In line with our previous studies (Lillis et al., 2012; Glykys et al., 2014), [Cl^–^]_i_ transiently increased in all pyramidal cells at the onset of spontaneous ILDs and was further elevated during the ILDs. Pharmacological manipulations of Cl transport could produce anticonvulsant effects that affect [Cl^–^]_i_ as a consequence of reduced seizure activity, separately from the consequences of altered transport. To estimate how direct anticonvulsant effects might alter [Cl^–^]_i_, we applied the sodium channel blocker TTX (1 μm). TTX rapidly abolished recurrent ILDs and corresponding [Cl^–^]_i_ transients (Fig. 1*E*), and significantly reduced the median baseline [Cl^–^]_i_ from 8.9 (7.15–11) ± 4.7 to 7.5 (5.1–10.8) ± 4.1 mm [*N* = 6 slices at DIV11–DIV16, *n* = 32 paired cells; Wilcoxon signed-rank test, sum, of negative ranks (*W*) = 474, *Z* = 3.92, *p* < 0.001; Fig. 1*F*], suggesting activity dependent baseline neuronal chloride accumulation.

The baseline [Cl^–^]_i_ in the CA1 pyramidal cell layer progressively decreased during the first week of organotypic slice incubation [*N* = 29 slices at DIV0–DIV28, nonlinear extreme curve fit: Y0 = 23.7 ± 2.36 (median ± SE), XC = 8.3 ± 1.1, A = −16 ± 2.3 M; *R*^2^ = 0.67; ANOVA: *F* = 115.3, *p* < 0.001; Fig. 1*G*]. This progressive decrease in baseline [Cl^–^]_i_ and corresponding negative shift in E_Cl_ during the first week of incubation correlated with a decrease in the number of acutely damaged cells with higher [Cl^–^]_i_ because of dissection (Berdichevsky et al., 2012; Dzhala et al., 2012) as well as with postnatal changes in CCC expression (Stein et al., 2004; Dzhala et al., 2005; Takayama and Inoue, 2010). Starting from the second week of incubation, the median baseline [Cl^–^]_i_ progressively increased with corresponding positive shifts in E_Cl_ and the increasing incidence, duration and frequency of spontaneous ILDs (Fig. 1*D*,*G*). Positive shifts in E_Cl_ and corresponding changes in GABA action are thought to contribute to facilitation of recurrent epileptiform discharges and resistance of ILDs to GABAergic anticonvulsants (Dzhala and Staley, 2003; Dzhala et al., 2010; Khazipov et al., 2015; Glykys et al., 2017). We therefore determined the contribution of KCC2 cotransporter activity to neuronal chloride elevation and extrusion rates, and facilitation of recurrent ILDs during epileptogenesis.

### The selective KCC2 inhibitor VU0463271 reduced chloride extrusion and increased duration of ILDs

VU0463271, a potent and selective inhibitor of the neuronal K^+^-Cl^–^ cotransporter, KCC2 (IC_50_ = 61 nm), exhibits >100-fold selectivity versus the NKCC1 (Delpire et al., 2012). Selective inhibition of KCC2 with VU0463271 led to hyperexcitability and epileptiform discharges in hippocampal slices exposed to low Mg^2+^ and also *in vivo* (Sivakumaran et al., 2015). We determined the contribution of KCC2 cotransporter activity to baseline [Cl^–^]_i_ elevation and extrusion rates during recurrent ILDs, and their correlation with the frequency, duration, and power of ILDs.

**Figure 2.**
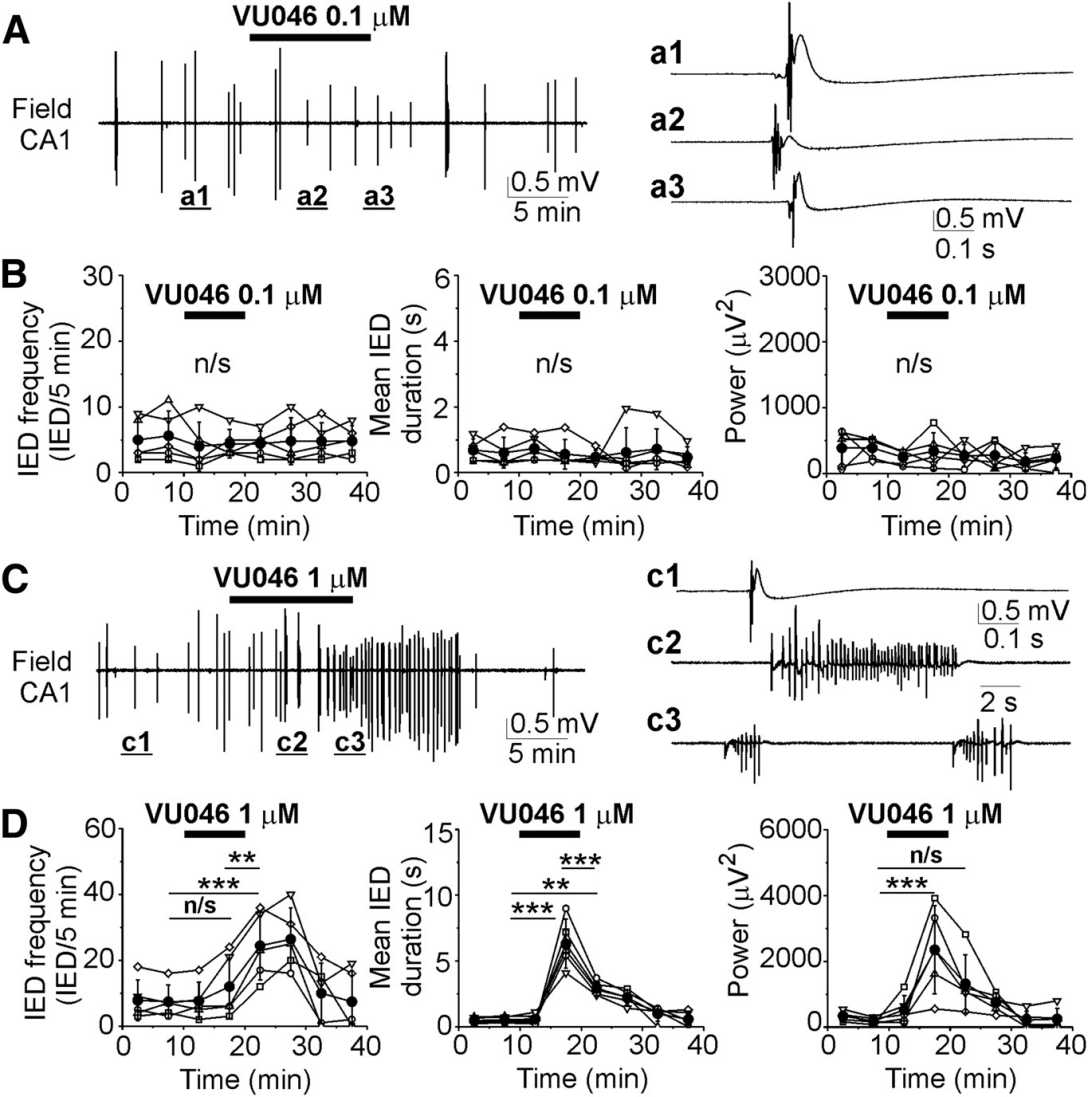
Effects of the KCC2 blocker VU0463271 on recurrent IEDs at DIV5–DIV7. ***A***, ***C***, Extracellular field potential recordings in the CA1 pyramidal cell layer in the organotypic hippocampal slices at DIV6 before (control), during and after application of 0.1 μm (***A***) and 1 μm (***C***) VU0463271 for 10 min. Expansion of IEDs in control and in the presence of VU0463271. ***B***, ***D***, Corresponding summary plots of the frequency and mean duration of recurrent IEDs in individual cultures (open symbols) and corresponding power of electrical activity in 5-min windows. Filled symbols indicate group mean ± SD. VU0463271 (1 μm) significantly increased the mean frequency, duration, and power of epileptiform discharges (n/s corresponds to *p* > 0.05; *p < 0.05; **p < 0.01; ***p < 0.001, one-way RM ANOVA, Tukey’s test).

**Figure 3.**
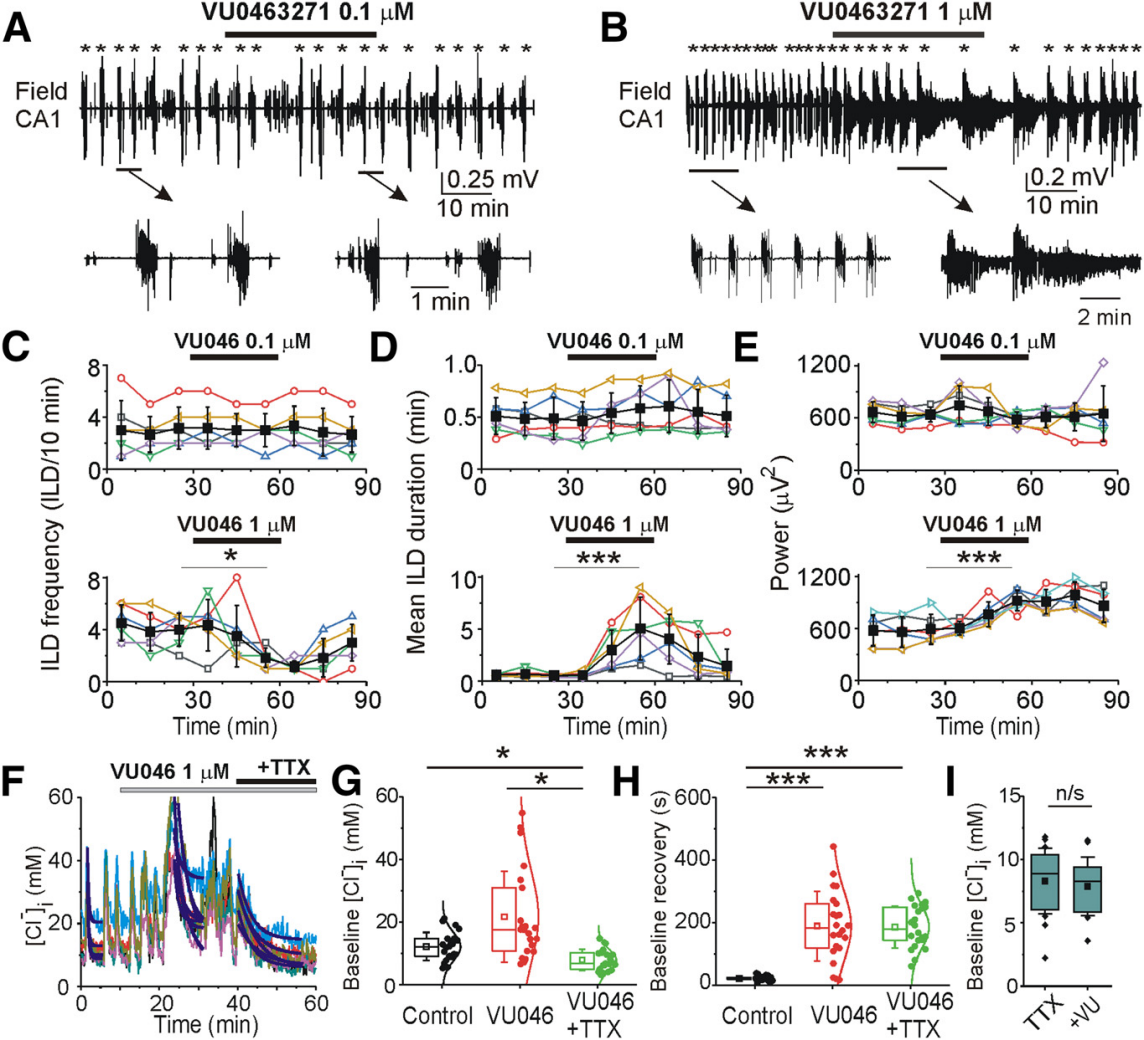
Effects of the KCC2 blocker VU0463271 on recurrent ILDs and intracellular chloride at DIV14–DIV21. ***A***, ***B***, Extracellular field potential recordings in the CA1 pyramidal cell layer in the organotypic hippocampal slices at DIV18 before (control), during and after application of 0.1 μm (***A***) and 1 μm (***B***) VU0463271 for 30 min. Expansion of ILDs in control and in the presence of VU0463271. ***C–E***, Corresponding summary plots of the frequency and mean duration of recurrent ILDs and corresponding power of electrical activity in 10-min windows in individual slice cultures (open symbols). Filled symbols indicate group mean ± SD. VU0463271 (1 μm) significantly reduced the mean frequency of ILDs and increased their duration and power (*corresponds to *p* < 0.05; ***p < 0.01, one-way RM ANOVA, Tukey’s test). ***F***, Exponential fit (y = A1exp(–(x – x0)/t1) + y0) was used to measure base line [Cl^–^]_i_ (y0) and decay time (t1) of base line [Cl^–^]_i_ recovery in individual cells (solid navy curves) during ILD in control, during application of VU0463271 and TTX (adj. *R*^2^ = 0.9–0.96). ***F–H***, VU0463271 (1 μm) increased base line [Cl^–^]_i_ (y0) and delayed decay time of baseline chloride recovery (t1) during ILDs. ***G***, TTX (1 μm) abolished ILDs and slowly reduced the median baseline [Cl^–^]_i_ (*p < 0.05, Friedman RM ANOVA on ranks, Tukey’s test). ***H***, Decay time of baseline [Cl^–^]_i_ recovery during application of TTX in the presence of VU0463271 was significantly higher as in control (***p < 0.001, one-way RM ANOVA, Tukey’s test). ***I***, Effects of VU0463271 in the presence of TTX on [Cl^–^]_i_. VU0463271 application in the presence of TTX did not significantly change the median baseline chloride concentration (*p* > 0.05, Wilcoxon signed-ranks test).

Extracellular field potential recordings were performed in the CA1 pyramidal cell layer in the organotypic hippocampal slices at DIV5–DIV7 (Fig. 2) and DIV14–DIV21 (Fig. 3) before (control), during and after application of VU0463271 (0.1 and 1 μm). At DIV5–DIV7, spontaneous neuronal activity was characterized by multiunit activity and population bursts reminiscent of interictal-like epileptiform discharges (IEDs) (Dyhrfjeld-Johnsen et al., 2010; Berdichevsky et al., 2012). Bath application of VU0463271 at low concentration (0.1 μm for 10 min) did not change the frequency (*N* = 5 slices, one-way RM ANOVA: DF = 39, *F* = 0.328, *p* = 0.934) and duration of IEDs (one-way RM ANOVA: DF = 39, *F* = 0.345, *p* = 0.926), nor the power of electrical activity in all pairwise 5-min windows (one-way RM ANOVA: DF = 39, *F* = 0.753, *p* = 0.63; Fig. 2*A*,*B*). VU0463271 at the higher concentration (1 μm for 10 min) significantly increased the frequency, duration and power of spontaneous IEDs (Fig. 2*C*,*D*). VU0463271 (1 μm) progressively increased the mean frequency of IEDs from 7.4 ± 5.12 IED/5 min in control to 12 ± 9.7 IEL/5 min in the presence of VU0463271 and then to 24.4 ± 10.45 IED/5 min during wash [*N* = 5; one-way RM ANOVA: DF = 39, *F* = 15.2, *p* < 0.001; Tukey’s test: VU04663271 (15–20 min) vs control (5–10 min), df = 4.6, *q* = 2.267, *p* = 0.745; wash (20–25 min) vs control (5–10 min), df = 17, *q* = 8.377, *p* < 0.001; wash (20–25 min) vs VU04663271 (15–20 min), df = 12.4, *q* = 6.11, *p* = 0.004; Fig. 2*D*]. VU0463271 (1 μm) significantly increased the corresponding group mean duration of IEDs from 0.5 ± 0.23 s in control to 6.3 ± 1.87 s in the presence of VU0463271 and then reduced to 2.9 ± 0.54 s during wash [*N* = 5; one-way RM ANOVA: DF = 39, *F* = 32.4, *p* < 0.001; Tukey’s test: VU04663271 (15–20 min) vs control (5–10 min), df = 7.79, *q* = 16.26, *p* < 0.001; wash (20–25 min) vs control (5–10 min), df = 2.41, *q* = 6.77, *p* = 0.001; wash (20–25 min) vs VU04663271 (15–20 min), df = −3.38, *q* = 9.49, *p* < 0.001; Fig. 2*D*]. The corresponding power of electrical activity in 5-min windows significantly increased from 183.2 ± 98.9 μV^2^ in control to 2352.2 ± 1339.9 μV^2^ in the presence of VU0463271 (15–20 min) and then reduced to 1323.2 ± 882.5 μV^2^ [*N* = 5; one-way RM ANOVA: DF = 39, *F* = 8.807, *p* < 0.001; Tukey’s test: VU04663271 (15–20 min) vs control (5–10 min), df = 2169, *q* = 8.54, *p* < 0.001; wash (20–25 min) vs control (5–10 min), df = 1139, *q* = 4.49, *p* = 0.062; wash (20–25 min) vs VU04663271 (15–20 min), df = −1028, *q* = 4.05, *p* = 0.12; Fig. 2*D*].

At DIV14–DIV21, spontaneous neuronal network activity was characterized by short inter-ILDs and prolonged ILDs, reminiscent of seizure-like activity *in vivo* (Fig. 3*A*,*B*). VU0463271 at the lower concentration (0.1 μm) did not significantly change the frequency (*N* = 6 slices, one-way RM ANOVA: DF = 53, *F* = 0.699, *p* = 0.69; Fig. 3*A*,*C*) and duration (one-way RM ANOVA: DF = 53, *F* = 1.198, *p* = 0.325; Fig. 3*D*) of recurrent ILDs, nor the power of electrical activity in 10-min windows (one-way RM ANOVA: DF = 53, *F* = 0.799, *p* = 0.607; Fig. 3*E*). VU0463271 at the higher concentration (1 μm) significantly reduced the mean frequency of recurrent ILDs from 4 ± 1.26 ILD/10 min in control to 1.83 ± 0.75 ILD/10 min during VU04663271 (1 μm) application [*N* = 6 slices; one-way RM ANOVA: DF = 53, *F* = 4.44, *p* < 0.001; Tukey’s test: VU04663271 (50–60 min) vs control (20–30 min), df = −2.17, *q* = 4.88, *p* = 0.032; Fig. 3*B*,*C*] and substantially increased the group mean duration of recurrent ILDs from 0.56 ± 0.3 to 5.5 ± 3 min (*N* = 6; one-way RM ANOVA: DF = 53, *F* = 8.91, *p* < 0.001; Tukey’s test: VU04663271 vs control, df = 4.9, *q* = 7.98, *p* < 0.001; Fig. 3*D*), resulting in SE. The corresponding power of electrical activity in 10-min windows significantly increased from 591.6 ± 171.7 to 920.2 ± 117.4 μV^2^ (*N* = 6, one-way RM ANOVA: DF = 53, *F* = 12.66, *p* < 0.001; Tukey’s test: VU04663271 vs control, df = 328.6, *q* = 6.83, *p* < 0.001; Fig. 3*E*).

**Figure 4.**
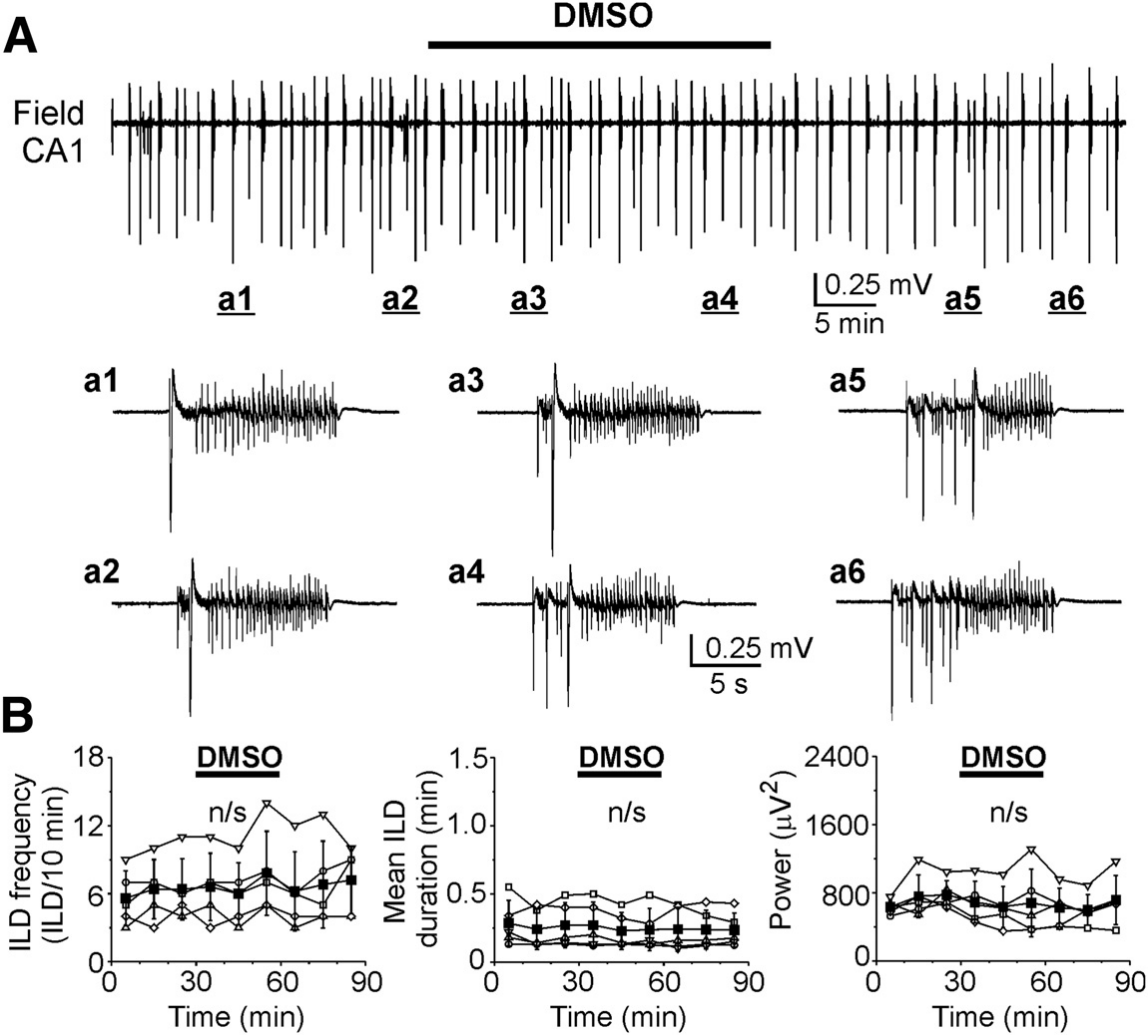
Effects of DMSO on recurrent ILDs at DIV12–DIhttV17. ***A***, Extracellular field potential recording in the CA1 pyramidal cell layer in the organotypic hippocampal slices at DIV14 before (control), during and after application of DMSO (100 μl/100 ml). Expansion of ILDs in control (***a1***, ***a2***), in the presence of DMSO (***a3***, ***a4***), and during washout of DMSO (***a5***, ***a6***). ***B***, Corresponding summary plots of the frequency and mean duration of recurrent ILDs and corresponding power of electrical activity in 10-min windows in individual slice cultures (open symbols). Filled symbols indicate group mean ± SD (n/s corresponds to *p* > 0.05, one-way RM ANOVA).

Under similar experimental conditions (DIV12–DIV17), bath application of dimethylsulfoxide (DMSO; 100 μl/100 ml), an organic solvent for VU04663271, did not change the frequency of ILDs (*N* = 5 slices, one-way RM ANOVA: DF = 44, *F* = 2.04, *p* = 0.073; Fig. 4*A*,*B*), duration of ILDs (*N* = 5, one-way RM ANOVA: DF = 44, *F* = 0.798, *p* = 0.61; Fig. 4*B*), and power of electrical activity in all pairwise 10-min windows (*N* = 5, one-way RM ANOVA: DF = 44, *F* = 1.141, *p* = 0.364; Fig. 4*B*). The increased duration and power of ILDs in the presence of VU04663271 (1 μm) suggest that KCC2 transport activity contributes to recovery of baseline [Cl^–^]_i_ and GABAergic inhibition during epileptiform discharges, and may contribute to termination of ILDs, although the concentration of antagonist required was substantially higher than the reported IC_50_ for KCC2 (Delpire et al., 2012).

To test whether these “dual” effects of VU04663271 (1 μm; a reduction of the frequency of recurrent ILDs, but an increase in the duration and power of ILDs) were associated with changes in neuronal baseline [Cl^–^]_i_ and chloride transport, we compared the baseline [Cl^–^]_i_ and decay time constant of [Cl^–^]_i_ extrusion during recurrent ILDs in control conditions, during application of VU04663271, and during consecutive application of the sodium channel blocker TTX in the presence of VU04663271 (Fig. 3*F–H*). TTX application blocks seizure activity directly, providing a means to assess the effects of the intensity of ILD activity on [Cl^–^]_i_ levels and kinetics separately from effects on Cl transport. Bath application of VU04663271 (1 μm for 30 min) increased the median baseline chloride concentration from 12.1 (9.2–14.7) ± 4.5 to 17.7 (10.8–31) ± 14.4 mm, and [Cl^–^]_i_ remained elevated during intermittent ILDs. Subsequent application of TTX (1 μm), in the continued presence of VU0463271, abolished ILDs and significantly reduced the median baseline [Cl^–^]_i_ to 6.9 (5–10.3) ± 3.3 mm [*N* = 4 slices at DIV14–DIV21, *n* = 24 paired cells; Friedman RM ANOVA on ranks: χ^2^ = 36.3, *p* < 0.001; Tukey’s test: control compared with VU0463271, difference of ranks (df) = 12, *q* = 2.6, *p* > 0.05; control vs TTX in the presence of VU0463271, df = 27, *q* = 5.8, *p* < 0.05; VU0463271 vs TTX in the presence of VU0463271, df = 39, *q* = 8.3, *p* < 0.05; Fig. 3*G*]. The corresponding decay time constant of baseline [Cl^–^]_i_ extrusion (recovery) during ILDs significantly increased from the mean 21.4 ± 7.6 s in control to 188.1 ± 111 s in the presence of VU04663271, and subsequent application of TTX in the presence of VU0463271 abolished [Cl^–^]_i_ transients and recovered baseline [Cl^–^]_i_ with a time constant of 185.5 ± 67.2 s [*N* = 4 slices at DIV14–DIV21, *n* = 24 paired cells; one-way RM ANOVA: difference of variations (DF) = 65, *F* = 38.6, *p* < 0.001; Tukey’s test: control compared with VU04663271, difference of means (df) = −166.7, *q* = 10.84, *p* < 0.001; control vs TTX in the presence of VU04663271, df = −164.1, *q* = 10.68, *p* < 0.001; VU04663271 vs TTX in the presence of VU04663271, df = −2.6, *q* = 0.166, *p* = 0.993; Fig. 3*H*]. In contrast, VU0463271 application in the presence of TTX (Fig. 3*I*) did not significantly change the median baseline chloride concentration from 8.5 (5.7–11.8) ± 2.7 to 8.3 (5.7–9.9) ± 2.4 mm (*N* = 4 slices at DIV14–DIV17, *n* = 24 paired cells; Wilcoxon signed-rank test: *W* = 200, *Z* = 1.41, *p* = 0.16; Fig. 3*I*). Thus, the increased duration and power of intermittent ILDs in the presence of the KCC2 blocker VU04663271 correlates with increased magnitude and duration of [Cl^–^]_i_ elevation, consistent with a reduced extrusion rate of [Cl^–^]_i_.

**Figure 5.**
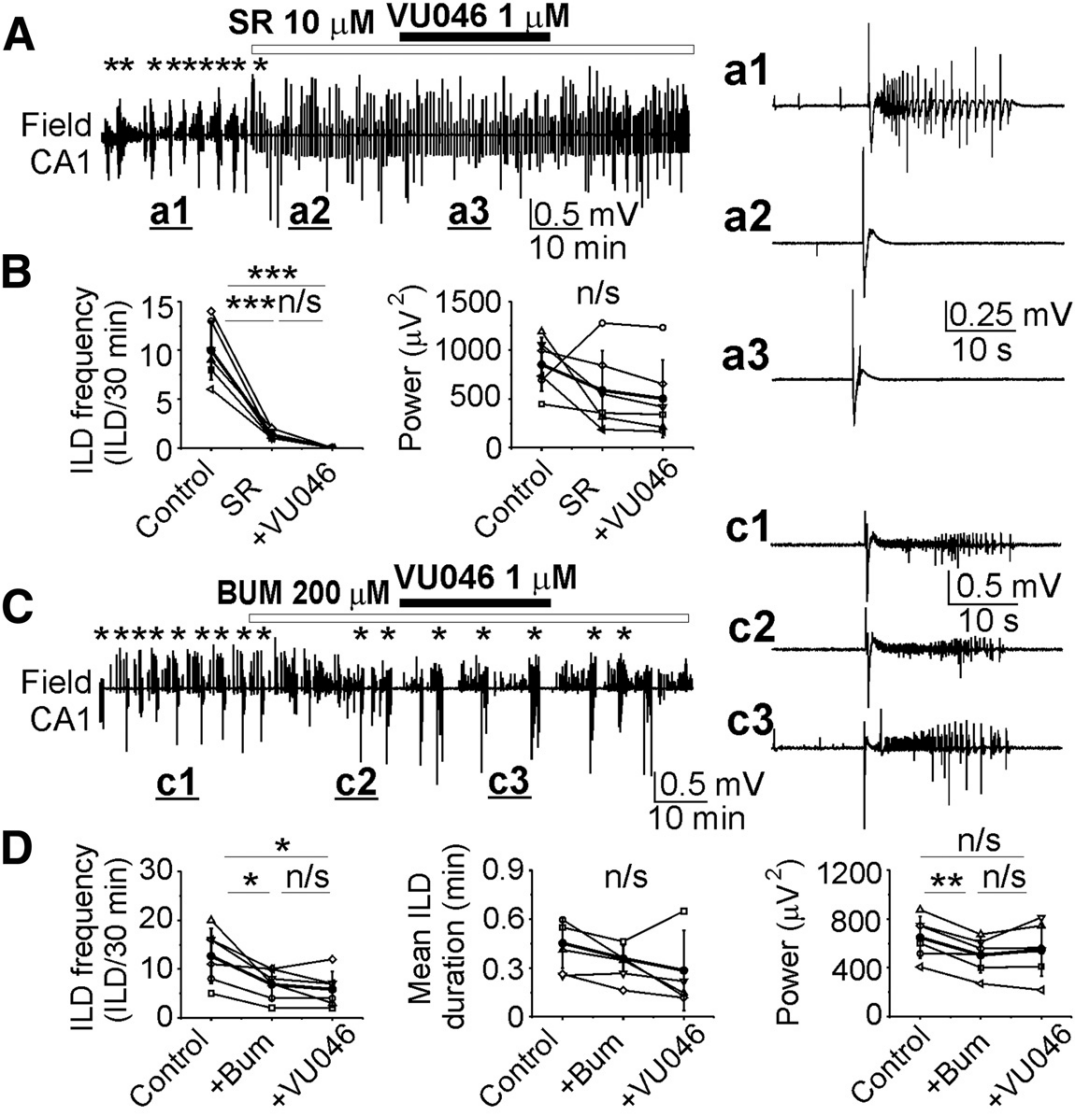
Selectivity of KCC2 blocker VU0463271. ***A***, ***C***, Extracellular field potential recordings in the CA1 pyramidal cell layer in the organotypic hippocampal slices *in vitro*. ***A***, ***B***, Application of the GABA_A_-R antagonist SR95531 (10 μm) abolished spontaneous ILDs and induced large amplitude IEDs. Subsequent application of VU0463271 (1 μm) in the presence of SR95531 did not change the mean frequency of ILDs discharges (n/s, *p* > 0.05; ***p < 0.001; one-way RM ANOVA, Tukey’s test) and power of electrical activity (*p* > 0.05; one-way RM ANOVA). ***A***, ***a1–a3***, Expansion of ILD in control (***a1***) and IEDs in the presence of SR95531 (***a2***) and VU0463271 (***a3***). ***C***, ***D***, Application of VU0463271 (1 μm) in the presence of NKCC1 and KCC2 blocker bumetanide (200 μm) did not change the mean frequency and duration of ILDs, and the mean power of electrical activity (*p* > 0.05, one-way RM ANOVA, Tukey’s test). ***c1–c3***, Expansion of ILDs in control (***c1***), during application of bumetanide (***c2***), and in the presence of VU0463271 (***c3***).

We next used pharmacological tools to determine whether GABA_A_-R block prevents the epileptogenic action of the KCC2 antagonist VU04663271 (Fig. 5*A*,*B*). Under similar experimental conditions (DIV14–DIV18), bath application of the GABA_A_-R antagonist SR95531 (10 μm) reduced the mean frequency of spontaneous ILDs from 10 ± 3 to 1.33 ± 0.52 ILD/30 min and induced large amplitude inter-ILDs (IEDs). Subsequent application of VU04663271 (1 μm), in the continued presence of SR95531, did not change the mean frequency of epileptiform discharges (*N* = 6 slices at DIV14–DIV18; one-way RM ANOVA: DF = 17, *F* = 59.85, *p* < 0.001; Tukey’s test: control compared with SR95531, df = 8.7, *q* = 12.3, *p* < 0.001; control compared with VU04663271 in the presence of SR95531, df = 10.0, *q* = 14.3, *p* < 0.001; SR95531 compared with VU04663271 in the presence of SR95531, df = 1.33, *q* = 1.9, *p* = 0.405; Fig. 5*B*). The net effect of the changes in ictal and interictal activity was a non-significant decrease in the mean power of electrical activity by 31%, from 852.5 ± 275.3 μV^2^ in control to 588.1 ± 408.32 μV^2^ in the presence of SR95531, and to 503.3 ± 396.6 μV^2^ during subsequent application of VU04663271 (1 μm), in the continued presence of SR95531 (*N* = 6; one-way RM ANOVA: DF = 17, *F* = 2.231, *p* = 0.16; Fig. 5*B*). These data suggest that the epileptogenic effects of the KCC2 blocker VU04663271 on the duration and power of ILDs require activation of GABA_A_ receptors.

We also tested whether cation-chloride cotransport was necessary for the epileptogenic effects of VU04663271 (Fig. 5*C*,*D*). Under similar experimental conditions (DIV14–DIV19), bath application of a concentration of bumetanide (200 μm) that blocks both NKCC1 and KCC2 cotransporters significantly reduced the mean frequency of spontaneous ILDs from 12.67 ± 5.6 to 6.8 ± 3.2 ILD/30 min. Subsequent application of 1uM VU04663271, in the continued presence of bumetanide, did not significantly change the mean frequency of ILDs to 5.8 ± 3.7 ILD/30 min (*N* = 6 slices at DIV14–DIV19; one-way RM ANOVA: DF = 17, *F* = 7.9, *p* = 0.009; Tukey’s test: control compared with bumetanide, df = 5.87, *q* = 4.4, *p* = 0.026; control vs VU04663271 in the presence of bumetanide, df = 6.87, *q* = 5.2, *p* = 0.011; bumetanide vs VU04663271 in the presence of bumetanide, df = 1, *q* = 76, *p* = 0.855; Fig. 5*D*). In addition, both bumetanide and subsequent VU04663271 applications did not change the mean duration of ILDs (*N* = 5 slices; one-way RM ANOVA: DF = 14, *F* = 2.4, *p* = 0.152; Fig. 5*D*). The net effect of the changes in ictal and interictal activity was a significant decrease in the mean power of electrical activity by 23% from 648.8 ± 172.4 μV^2^ in control to 504.17 ± 147.3 μV^2^ in the presence of bumetanide (Fig. 5*D*). Subsequent application of 1 μm VU04663271, in the continued presence of bumetanide, did not significantly change the mean power of electrical activity to 547.6 ± 218.1 μV^2^ (*N* = 6; one-way RM ANOVA: DF = 17, *F* = 7.6, *p* = 0.01; Tukey’s test: control compared with bumetanide, df = 144.7, *q* = 5.36, *p* = 0.009; control vs VU04663271 in the presence of bumetanide, df = 101.17, *q* = 3.75, *p* = 0.06; bumetanide vs VU04663271 in the presence of bumetanide, df = −43.5, *q* = 1.62, *p* = 0.51; Fig. 5*D*). These results suggest that KCC2 transporter activity is functional during recurrent ILDs, that VU04663271 antagonizes KCC2, and that this antagonism is necessary for the observed increase in the duration and power of ILDs, and induction of electrical SE (Fig. 3).

### Furosemide suppressed recurrent ILDs and delayed chloride extrusion

**Figure 6.**
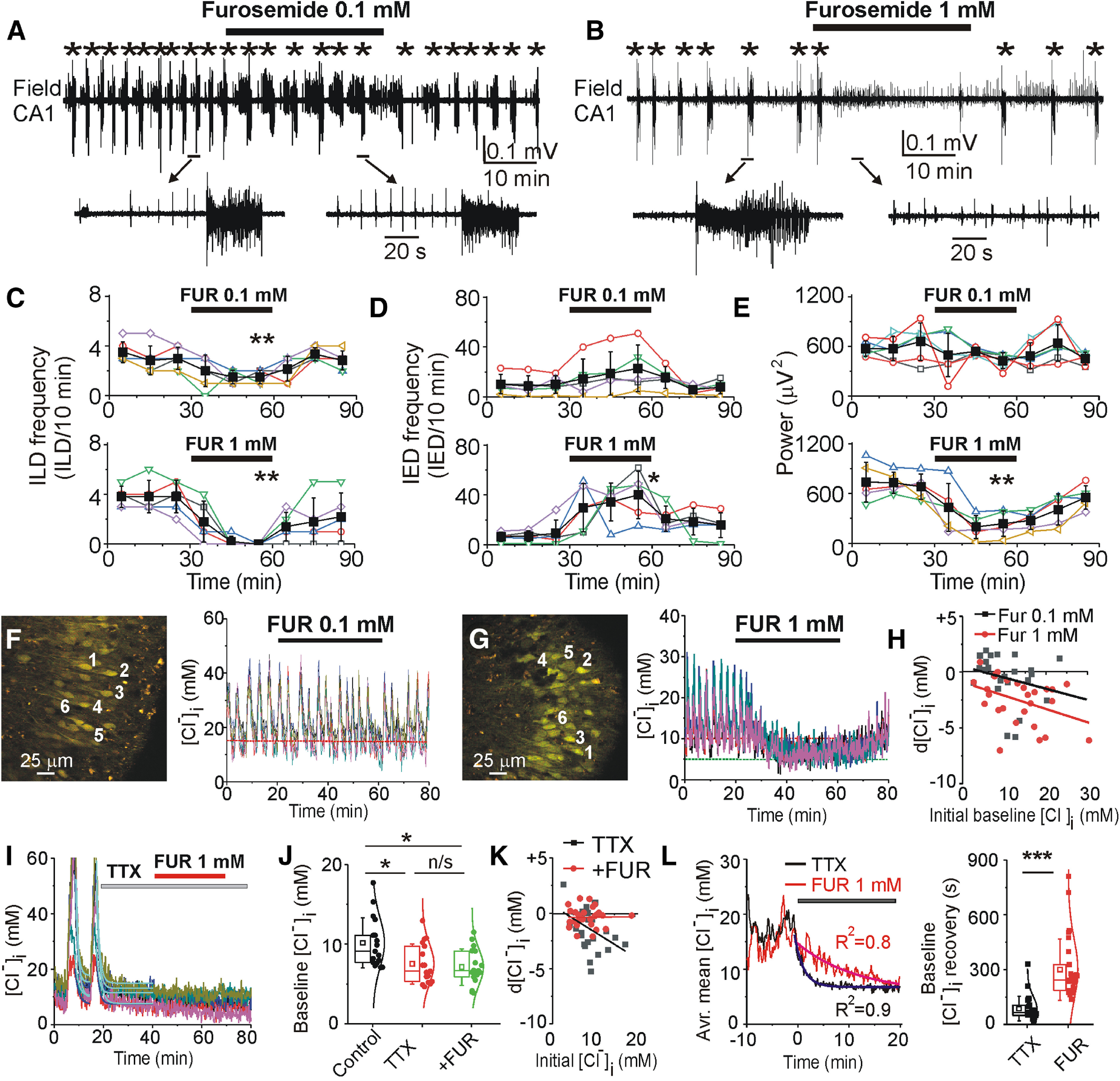
Effects of NKCC1/KCC2 blocker furosemide on recurrent epileptiform discharges and [Cl^–^]_i_. ***A***, ***B***, Extracellular field potential recording in the CA1 pyramidal cell layer in the organotypic hippocampal slice at DIV22 before (control), during and after application of furosemide (0.1 and 1 mm for 30 min). ***C–E***, Summary plots of the frequency of ILDs, IEDs, and power of corresponding activity in 10-min windows in individual slice cultures (open symbols). Filled symbols indicate mean ± SD. Furosemide (0.1 mm) reduced the frequency of ILDs and increased the frequency of IEDs. Furosemide (1 mm) abolished recurrent ILDs, increased the frequency of IEDs, and significantly decreased the power of electrical activity (*corresponds to *p* < 0.05; **p < 0.01, paired sample *t* test). ***F–H***, Effects of furosemide (0.1 and 1 mm) on [Cl^–^]_i_. Furosemide (1 mm) abolished recurrent ILDs and corresponding [Cl^–^]_i_ transients, and progressively reduced the baseline [Cl^–^]_i_. ***H***, Baseline [Cl^–^]_i_ changes in the presence of 0.1 mm furosemide and 1 mm furosemide as a function of an initial baseline [Cl^–^]_i_. ***I–K***, Effects of TTX (1 μm) and furosemide (1 mm) in the presence of TTX on [Cl^–^]_i_. TTX rapidly abolished chloride transients, reduced the median baseline [Cl^–^]_i_ (*p < 0.05 compared with control, Friedman RM ANOVA on ranks, Tukey’s test) and prevented effects of furosemide on [Cl^–^]_i_ (*p* > 0.05 compared with TTX). ***L***, Averaged mean [Cl^–^]_i_ changes before and after applications of TTX (black) or furosemide (red). Data were fitted with exponential fit to calculate decay time of [Cl^–^]_i_ extrusion during suppression of ILDs. Box (left) + data (right) plots correspond to median (25−75%) [Cl^–^]_i_ in paired cells (filled circles) and their distribution curves; open squares and whisker range indicate mean ± SD. Furosemide significantly delayed chloride extrusion during suppression of ILDs (***p < 0.001; Mann–Whitney rank-sum test).

**Figure 7.**
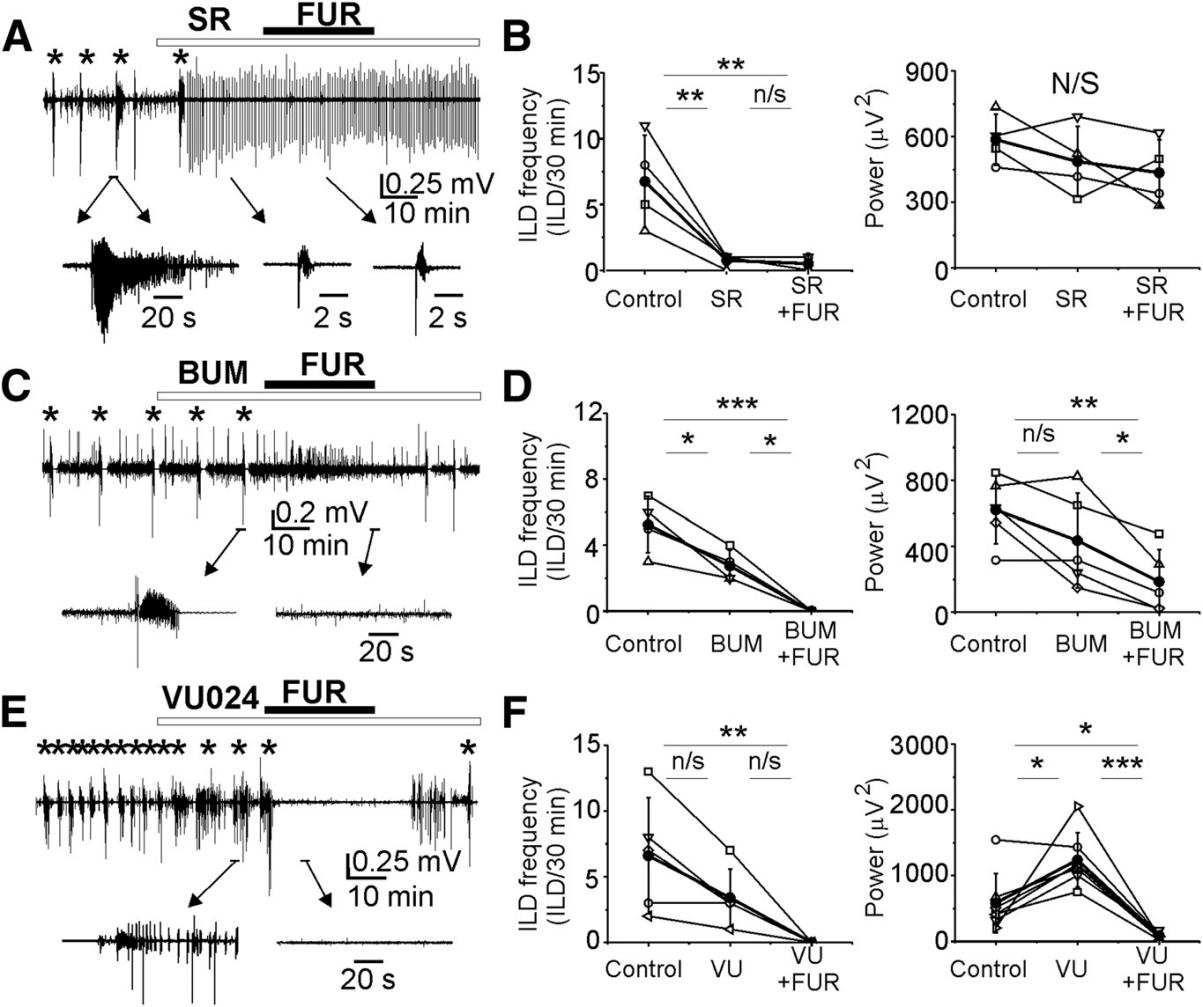
GABA_A_ receptor-dependent anti-ictal effects of furosemide was enhanced in the presence of NKCC1 and KCC2 antagonists. ***A***, ***C***, ***E***, Extracellular field potential recordings in the CA1 pyramidal cell layer in the organotypic hippocampal slices *in vitro*. Furosemide (1 mm) was applied in the presence of (***A***) GABA_A_ receptor antagonist SR95531 (10 μm), (***C***) NKCC1 blocker bumetanide (10 μm), and (***E***) KCC2 blocker VU0240551 (10 μm). ***A***, ***B***, SR95531 abolished recurrent ILDs, induced hypersynchronous interictal bursts, and prevented the anti-ictal effect of furosemide. ***C***, ***D***, Furosemide potentiated anticonvulsant effect of bumetanide (10 μm). ***E***, ***F***, Furosemide in the presence of VU0240551 abolished ictal and IEDs. ***B***, ***D***, ***F***, Corresponding summary data of the frequency of recurrent ILDs and power of electrical activity before and during drugs applications. Anti-ictal effects of furosemide were significant in the presence of bumetanide (***D***) and VU0240551 (***F***). *N* = 5–6 slices in each group of experiments; *p < 0.05, **p < 0.01, ***p < 0.001; one-way RM ANOVA, Tukey’s test.

The loop diuretic furosemide acts to inhibit both NKCC and KCC with about equal potency (K_i_ = 25–50 μm). In contrast to the epileptogenic action of the KCC2 inhibitor VU0463271 (Figs. 2, 3), high concentrations of furosemide (1–2 mm) demonstrate strong anti-ictal effects in various *in vitro* models of epilepsy (Hochman et al., 1995; Gutschmidt et al., 1999; Haglund and Hochman, 2005; Blauwblomme et al., 2018). To address these discrepancies, we determined the effects of furosemide (0.1 mm and 1 mm) on spontaneous epileptiform discharges alone and in combination with GABA_A_ receptor inhibitor SR95531, NKCC1 blocker Bumetanide and the low-affinity KCC2 blocker VU0240551 (Figs. 6, 7).

Control extracellular field potential recordings in the CA1 pyramidal cell layer revealed spontaneous IEDs and ILDs in all hippocampal slice cultures at DIV16–DIV19. Furosemide at low concentration (0.1 mm) significantly decreased the mean frequency of ILDs from 3 ± 0.89 to 1.5 ± 0.55 ILD/10 min (*N* = 6 slices; paired sample *t* test, *t* = 4.39, df = 5, *p* = 0.007; Fig. 6*A*,*C*) and increased the mean frequency of IEDs from 10 ± 7.1 to 22.8 ± 18.6 IED/10 min (paired sample *t* test, *t* = −1.94, df = 4, *p* = 0.12; Fig. 6*D*). The net effect of the changes in ictal and interictal activity was a decrease in the mean power of electrical activity by 35%, from 656.8 ± 222.9 to 427.7 ± 93.6 μV^2^ (paired sample *t* test, *t* = 1.92, df = 5, *p* = 0.11; Fig. 6*E*). Furosemide at high concentration (1 mm) abolished ILDs during 30 min of application (*N* = 5 slices; paired sample *t* test, *t* = 6.51, df = 4, *p* = 0.003; Fig. 6*C*) but significantly increased the mean frequency of IEDs from 9.2 ± 10.7 to 40 ± 19 IED/10 min (*N* = 5 slices; paired sample *t* test, *t* = −3.45, df = 4, *p* = 0.023; Fig. 6*D*). These changes resulted in a net decrease in the mean power of electrical activity by 65%, from 682.1 ± 153.1 to 236.7 ± 152.6 μV^2^ (*N* = 5; paired sample *t* test, *t* = 5.7, df = 4, *p* = 0.004; Fig. 6*D*).

We next determined whether the anticonvulsant effects of furosemide correlate with changes in baseline [Cl^–^]_i_ and the rate of [Cl^–^]_i_ extrusion during suppression of recurrent ILDs (Fig. 6*F–L*). Bath application of low concentrations of furosemide (0.1 mm) did not significantly change the median baseline [Cl^–^]_i_ between ILDs from 9.6 (5.8–13.4) ± 5.3 to 8.3 (6.54–13.75)±5.24 mm (*N* = 4 slices, *n* = 27 paired cells; paired sample Wilcoxon signed-rank test: *W* = 167, *Z* = 0.47, *p* = 0.64; Fig. 6*F*,*H*). High concentrations of furosemide (1 mm) abolished recurrent ILDs and corresponding [Cl^–^]_i_ transients and significantly reduced the median baseline [Cl^–^]_i_ from 12.8 (7.7–17.3) ± 5.9 to 9.9 (5.2–13.6) ± 5.6 mm (*N* = 4 slices at DIV13–DIV17, *n* = 27 paired cells; paired sample Wilcoxon signed-rank test: *W* = 347, *Z* = 4.34, *p* < 0.001; Fig. 6*G*,*H*). We also compared the effects of furosemide (1 mm) on baseline [Cl^–^]_i_ and the postictal decay time constant of [Cl^–^]_i_ extrusion with the corresponding effects of TTX, and furosemide in the presence of TTX (Fig. 6*I–L*). The sodium channel blocker TTX (1 μm) rapidly abolished recurrent ILDs and significantly reduced the median baseline [Cl^–^]_i_ from 9.1 (7.67–11.6) ± 3.1 to 6.6 (5.3–9.9) ± 2.5 mm, and subsequent application of furosemide (1 mm) in the presence of TTX did not change the median baseline [Cl^–^]_i_ to 6.7 (5.8–9.1) ± 2.3 mm (*N* = 4 slices at DIV13–DIV17, *n* = 17 paired cells; Friedman RM ANOVA on ranks: χ^2^ = 13.2, *p* = 0.001; Tukey’s test: control compared with TTX, df = 16, *q* = 3.96, *p* < 0.05; control compared with furosemide in the presence of TTX, df = 20, *q* = 4.85, *p* < 0.05; TTX compared with furosemide in the presence of TTX, df = 4, *q* = 0.97, *p* > 0.05; Fig. 6*J*). The median decay time constant of chloride extrusion during TTX application and suppression of ILDs was 65 (48.9–112.7) ± 66.9 s (*N* = 5 slices at DIV14–DIV17, *n* = 30 cells; Fig. 6*L*), significantly lower compared with corresponding value 242.7 (187–328.1) ± 167.9 s (*N* = 5 slices at DIV14–DIV17, *n* = 31 cells; Fig. 6*L*) during furosemide application (Mann–Whitney rank-sum test: U = 32, Z=−5.67, *p* < 0.001). The reduction in postictal [Cl^–^]_i_ extrusion by high concentrations of furosemide was greater than the reduction induced by the KCC2-specific blocker VU0463271 (Fig. 3*H*). These data also suggest that the anti-ictal effects of high concentrations of furosemide are because of interactions with an unidentified Cl^–^ transporter, exchanger, or channel.

We next used pharmacological tools to determine whether GABA_A_-R and CCC (NKCC1 and KCC2) inhibitors prevent the anti-ictal effects of furosemide (Fig. 7). Under similar experimental conditions, bath application of furosemide (1 mm) in the presence of the GABA_A_ receptor antagonist SR95531 (10 μm) did not change the mean frequency of epileptiform discharges (*N* = 4 slices; one-way RM ANOVA: DF = 11, *F* = 15.7, *p* = 0.004; Tukey’s test: control compared with SR95531, df = 6, *q* = 6.71, *p* = 0.008; control vs furosemide in the presence of SR95531, df = 6.25, *q* = 6.99, *p* = 0.006; SR95531 vs furosemide in the presence of SR95531, df = 0.25, *q* = 0.28, *p* = 0.979; Fig. 7*A*,*B*) and the mean power of epileptiform discharges (one-way RM ANOVA: DF = 11, *F* = 1.48, *p* = 0.29). In contrast, furosemide (1 mm) strongly enhanced the anticonvulsant action of bumetanide applied at a concentration (10 μm) that selectively blocked NKCC1 (Fig. 7*C*,*D*). Initial application of bumetanide (10 μm) significantly reduced the mean frequency of ILDs from 5.25 ± 1.7 to 2.75 ± 0.96 ILD/30 min, and subsequent application of furosemide (1 mm) in the presence of bumetanide abolished recurrent ILDs (*N* = 4 slices; one-way RM ANOVA: DF = 11, *F* = 30.1, *p* < 0.001; Tukey’s test: control vs bumetanide, df = 2.5, *q* = 5.22, *p* = 0.024; control vs furosemide in the presence of bumetanide: df = 5.25, *q* = 10.9, *p* < 0.001; bumetanide vs furosemide in the presence of bumetanide: df = 2.75, *q* = 5.75, *p* = 0.016; Fig. 7*C*,*D*). Corresponding power of electrical activity decreased from 620.9 ± 206.3 μV^2^ in control to 435.2 ± 287.7 μV^2^ during bumetanide application, and to 185.1 ± 196.6 μV^2^ during subsequent application of furosemide in the presence of bumetanide (one-way RM ANOVA: DF = 14, *F* = 14.7, *p* = 0.002; Tukey’s test: control vs bumetanide, df = 185.7, *q* = 3.26, *p* = 0.113; control vs furosemide in the presence of bumetanide: df = 435.8, *q* = 7.6, *p* = 0.002; bumetanide vs furosemide in the presence of bumetanide: df = 250.1, *q* = 4.39, *p* = 0.035; Fig. 7*C*,*D*).

Under similar experimental conditions (DIV16–DIV19), bath application of the low-affinity KCC2 antagonist VU0240551 (10 μm for 30 min) reduced the mean frequency of ILDs from 6.6 ± 4.4 to 3.4 ± 2.2 ILD/30 min and increased the frequency of IEDs, and consecutive application of furosemide (1 mm) in the presence of VU0240551 (10 μm) rapidly abolished both ILDs and IEDs (*N* = 5; one-way RM ANOVA: DF = 14, *F* = 10.61, *p* = 0.006; Tukey’s test: control vs VU0240551, df = 3.2, *q* = 3.15, *p* = 0.125; control vs furosemide in the presence of VU0240551, df = 6.6, *q* = 6.514, *p* = 0.005; VU0240551 vs furosemide in the presence of VU0240551, df = 3.4, *q* = 3.36, *p* = 0.102; Fig. 7*E*,*F*). Corresponding power of electrical activity increased from 584.4 ± 448.7 μV^2^ in control to 1241 ± 415.9 μV^2^ during VU0240551 application, and consecutive application of furosemide (1 mm) in the presence of VU0240551 (10 μm) decreased the power of electrical activity to 87.7 ± 44.6 μV^2^ (one-way RM ANOVA: DF = 20, *F* = 19.2, *p* < 0.001; Tukey’s test: control vs VU0240551, df = −656.6, *q* = 4.97, *p* = 0.011; control vs furosemide in the presence of VU0240551, df = 496.7, *q* = 3.76, *p* = 0.041; VU0240551 vs furosemide in the presence of VU0240551, df = 1153.2, *q* = 8.7, *p* < 0.001; Fig. 7*E*,*F*). These results suggest that the anti-ictal action of furosemide requires functional GABA_A_ receptors, is independent of NKCC1 activity, and greatly exceeds the anti-ictal effects of specific blockers of KCC2 transport activity. Curiously, both bumetanide and VU0240551 strongly potentiated the antiepileptic action of furosemide. These results are consistent with anti-ictal effects arising from interactions with an unidentified Cl transporter, exchanger, or channel.

### Effects of KCC2 cotransporter enhancer CLP257 on [Cl^–^]_i_ and recurrent ILDs

Enhancing KCC2 transport activity may be a useful therapeutic strategy to restore and/or reduce a baseline [Cl^–^]_i_, improve GABAergic inhibition, suppress ILDs and prevent epileptogenesis (Gagnon et al., 2013; Moore et al., 2018). We therefore determined the acute anticonvulsant efficacy of the putative KCC2 activator CLP 257 (1–30 μm; Gagnon et al., 2013) and its effects on neuronal [Cl^–^]_i_ in the organotypic hippocampal slices model of epileptogenesis *in vitro*.

**Figure 8.**
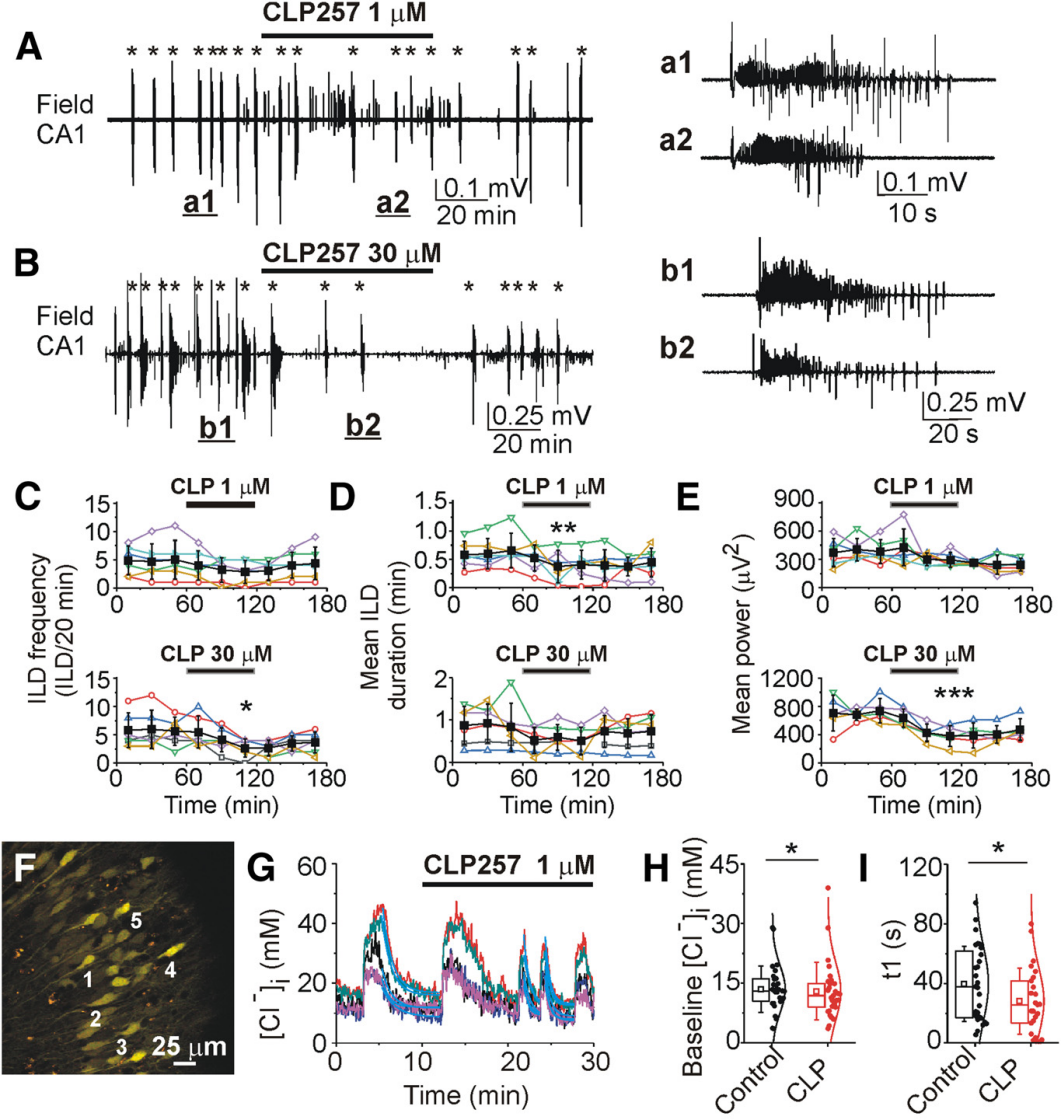
The KCC2 enhancer CLP257 reduced duration of ILDs. ***A***, ***B***, Extracellular field potential recordings in the CA1 pyramidal cell layer before (control), during and after application of CLP257 (1 and 30 μm for 1 h). Examples of ictal-like events in control and during application of 1 μm CLP257 (***A***, ***a1***, ***a2***) and 30 μm CLP257 (***B***, ***b1***, ***b2***). ***C–E***, Summary plots of the frequency and mean duration of ILDs, and power of electrical activity in individual slices (open symbols) and corresponding group mean ± SD (filled symbols) before, during and after 1 μm (top plots) and 30 μm (bottom plots) CLP257 applications. CLP257 (1 μm) significantly reduced the mean duration of ILDs (*p < 0.05, **p < 0.01, ***p < 0.001; paired sample *t* test). ***F***, Effects of CLP257 on [Cl^–^]_i_ as a function of time in *n* = 5 individual cell. ***G***, ***H***, Statistical histograms showing the effects of CLP257 (1 μm) on baseline [Cl^–^]_i_ and decay time of chloride extrusion (*p < 0.05, Wilcoxon signed-rank test). CLP257 (1 μm) significantly accelerated extrusion rate (t1) of neuronal chloride during recurrent ILDs.

CLP257 at low concentration (1 μm for 60 min) non-significantly decreased the mean frequency of ILDs from 5 ± 3.4 to 2.8 ± 2 ILD/20 min (*N* = 6 slices at DIV14–DIV20; paired sample *t* test, *t* = 1.73, df = 5, *p* = 0.14; Fig. 8*A*,*C*) and significantly reduced the group mean duration of ILDs from 0.66 ± 0.3 to 0.41 ± 0.26 min (paired sample *t* test, *t* = 4.19, df = 5, *p* = 0.008; Fig. 8*D*). The net effect of the changes in extracellular field potential activity was a decrease in the mean power in 20-min windows from 382.1 ± 125.3 to 293.9 ± 80.3 μV^2^ (paired sample *t* test, *t* = 2.42, df = 5, *p* = 0.059; Fig. 8*E*). CLP257 at high concentration (30 μm for 60 min) significantly decreased the mean frequency of ILDs from 5.67 ± 2.5 to 2.67 ± 1.63 ILD/20 min (*N* = 6 slices at DIV15–DIV20; paired sample *t* test, *t* = 3.5, df = 5, *p* = 0.017; Fig. 8*B*,*C*) and reduced the group mean duration of ILDs from 0.83 ± 0.57 to 0.59 ± 0.3 min (paired sample *t* test, *t* = 1.3, df = 5, *p* = 0.25; Fig. 8*D*). These changes resulted in a significant net decrease in the mean power of electrical activity in 20-min windows from 738.5 ± 171.3 to 380.2 ± 147.8 μV^2^ (paired sample *t* test, *t* = 11.5, df = 4, *p* < 0.001; Fig. 8*E*). In contrast to these effects of CLP257 on spontaneous epileptiform activity, a higher concentration of CLP257 (100 μm) increased the duration of ILDs in the 4-AP-induced model of epileptogenesis (Hamidi and Avoli, 2015), suggesting non-specific effects at higher concentrations of CLP257.

We next determined the effects of CLP257 on baseline [Cl^–^]_i_ and the postictal decay time constant of [Cl^–^]_i_ extrusion during recurrent ILDs (Fig. 8*F–I*). Bath application of CLP257 (1 μm for 30–40 min) induced a significant reduction in the median baseline [Cl^–^]_i_ between ILDs from 13.1 (10.3–16.1) ± 5.8 to 11.9 (8.8–15) ± 7.2 mm (*N* = 5 slices, *n* = 30 paired cells; Wilcoxon signed-rank test: *W* = 308, *Z* = 2.38, *p* = 0.017; Fig. 8*G*,*H*). In addition, CLP257 (1 μm) significantly reduced the median decay time constant of Cl^–^ extrusion during postictal recovery of baseline [Cl^–^]_i_ from 34.95 (16.87–58.15) ± 25.14 to 23.6 (7.89–40.65) ± 22.16 s (Wilcoxon signed-rank test: *W* = 238, *Z* = 2.5, *p* = 0.012; Fig. 8*I*). This reduction was correlated with a reduced duration of recurrent ILDs (Fig. 8*A*,*D*).

In conclusion, our results demonstrated that low concentration of CLP257 (1 μm) modestly improved [Cl^–^]_i_ homeostasis and reduced the duration of recurrent ILDs. However, CLP257 (1 μm) did not change the frequency of recurrent ILDs. In contrast, CLP257 at high concentration (30 μm) significantly reduced the frequency of ILDs and power of electrical activity.

## Discussion

### Summary of findings

In an *in vitro* preparation exhibiting spontaneous electrographic interictal spikes and ILDs, we found two important effects that were clearly not related to CCCs: (1) GABA_A_ receptor antagonists blocked ILDs and produced high-amplitude periodic inter-ILDs; (2) blocking ILDs reduced the baseline (steady state) [Cl^–^]_i_ independently of effects on CCCs. Manipulation of cation-chloride cotransport in this preparation with currently available pharmacological agents demonstrated: (3) antagonizing KCC2 activity prolonged the ictal increase in [Cl^–^]_i_; (4) antagonizing KCC2 activity increased ILD duration; (5) increasing KCC2 activity reduced the ictal [Cl^–^]_i_ increase; (6) increasing KCC2 activity reduced ILD durations; (7) the effects of high-affinity KCC2 modulators were dependent on the activity of GABA_A_ receptors; however, (8) the effects of the high-affinity KCC2 antagonist VU0463271 on [Cl^–^]_i_ and ILDs were only apparent at concentrations 20 times higher than the IC_50_ (61 nm) for KCC2 block established in dissociated cell cultures; (9) the effects of the KCC2 enhancer CLP257 on [Cl^–^]_i_ and ILDs were evident at two times the EC_50_ (616 nm) for KCC2 enhancement.

We also found evidence of effects of CCC antagonists that were not mediated by KCC2 or NKCC1. The largest of these effects was the profound GABA_A_ receptor-dependent anti-ictal effect of high concentrations of furosemide (Hochman et al., 1995; Gutschmidt et al., 1999; Haglund and Hochman, 2005; Blauwblomme et al., 2018) that was enhanced in the presence of NKCC1 and KCC2 antagonists (Fig. 7). This effect was correlated with a prolongation of the postictal [Cl^–^]_i_ transient that greatly exceeded the maximum effect of specific CCC blockers. These findings raise the possibility that blockade of an unidentified CCC, perhaps KCC3, mediates these remarkable anticonvulsant effects, but it is equally possible that the anticonvulsant effects are not related to the reduced Cl^–^ transport. Rather, other nonspecific effects (Andreasen and Nedergaard, 2017) may be responsible for this profound anticonvulsant effect, for example, interference with vesicular glutamate uptake (Roseth et al., 1995), or other membrane Cl^–^ exchangers or channels, particularly GABA_A_ receptor-operated channels in light of our first finding (Korpi and Lüddens, 1997; Mtchedlishvili and Kapur, 2006).

### The role of KCC2 transport activity in termination of ILDs

Activity-dependent neuronal Cl^–^ accumulation and a consequent transient depolarizing shift in the reversal potential of the GABA_A_ -R (E_GABA_) have been suggested to contribute to generation of ictal-clonic afterdischarges (Fujiwara-Tsukamoto et al., 2006, 2010; Ellender et al., 2014). We determined the role of KCC2 transport activity in extrusion of neuronal Cl^–^ and termination of ictal-like tonic-clonic activity using pharmacological inhibition of KCC2 transport activity. Our data demonstrated that the high-affinity KCC2 inhibitor VU0463271 increased ILD-induced [Cl^–^]_i_ elevation and the duration of ILDs (Fig. 3). Neuronal baseline Cl^–^ remained elevated during prolonged ictal-like activity and the mean decay time of chloride extrusion increased from the mean 21.4 ± 7.6 s in control to 188.1 ± 111 s in the presence of VU04663271. The sodium channel blocker TTX in the presence of VU0463271 abolished recurrent ILDs and recovered baseline [Cl^–^]_i_ within the mean 185.5 ± 67.2 s. In addition, VU0463271 application in the presence of TTX did not significantly change the mean baseline [Cl^–^]_i_. Our results demonstrate that KCC2 transport activity efficiently recovers E_Cl_ during ILDs, restores GABAergic inhibition and these effects are correlated with more rapid termination of ILDs. Inhibition of KCC2 transport activity delays chloride extrusion rate suggesting that impaired KCC2 function may contribute to the transition from the short ictal-like events to sustained SE.

### Modulation of CCCs for control of drug-resistant ILDs

Neuronal chloride concentration ([Cl^–^]_i_) is an important determinant of both postsynaptic GABA_A_-receptor-mediated signaling and cell volume regulation. Cl^–^ equilibrium is mediated by a Donnan system that includes intra and extracellular impermeant anions, and in which the CCCs comprise the requisite cation and chloride membrane permeability (Glykys et al., 2017). Increasing this permeability by increasing the maximum velocity of cation-chloride cotransport should not change the baseline [Cl^–^]_i_. However, increasing the maximum velocity of transport could increase the ability of neurons to buffer synaptically-mediated Cl^–^ influx. Importantly, this increased ability is predicated on the extracellular potassium; increases in extracellular potassium directly alter the steady state [Cl^–^]_i_.

The maximum velocity of CCCs is regulated by a system of the WNK-SPAK/OSR1 kinase complex pathways [serine-threonine kinase WNK (with no lysine) and SPS1-related proline/alanine-rich kinase (SPAK) or the SPAK homolog oxidative stress-responsive kinase 1 (OSR1)] that result in the compensatory phosphorylation and dephosphorylation processes (Kahle et al., 2006; Melo et al., 2013; Alessi et al., 2014; de Los et al., 2014). In immature neurons and isotonic conditions KCC2 is phosphorylated at two C-terminal threonine (Thr906 and Thr1007) and inactive, whereas CNS development and hypotonic conditions promote their dephosphorylation and activation (Kahle et al., 2013; Pisella et al., 2019; Watanabe et al., 2019).

Traumatic injury to the brain alters the equilibrium value of chloride, resulting in an elevated baseline [Cl^–^]_i_ and depolarizing shift in E_GABA_ (van den Pol et al., 1996; Pond et al., 2006; Kahle et al., 2008; Blaesse et al., 2009; Dzhala et al., 2010, 2012). Acute brain injury results in cytotoxic edema, in which there is a net neuronal uptake of chloride salts and water. Suppressing CCC activity may be a useful therapeutic strategy in this condition to prevent [Cl^–^]_i_ accumulation, reduce [Cl^–^]_i_ and swelling in injured neurons, restore GABAergic inhibition and suppress acute ILDs (Kahle et al., 2008; Gagnon et al., 2013; Glykys et al., 2017). Previous studies in *in vitro* and *in vivo* models of hypoxia-ischemia, recurrent seizures and neuronal brain injury demonstrated that inhibition of NKCC1 reduced [Cl^–^]_i_ in injured neurons, enhanced GABAergic inhibition and enhanced efficacy of GABAergic anticonvulsants (Dzhala et al., 2005, 2008, 2012; Pond et al., 2006; Nardou et al., 2009, 2011; Cleary et al., 2013; Dzhala and Staley, 2015; Sivakumaran and Maguire, 2016).

In the current study, we investigated the role of CCC inhibitors in chronic epilepsy *in vitro* rather than seizures in the setting of acute brain injury *in vivo*. Studies in acute brain slice preparations from patients with chronic epilepsy have supported the acute efficacy of bumetanide (Pallud et al., 2014). However, in acute brain slices, there is also a significant degree of acute brain injury (Dzhala et al., 2012), making it difficult to separate effects because of acute versus chronic changes. We found only GABA_A_ receptor-dependent pro-ictal effects of KCC2 antagonists at concentrations that also prolonged ictal [Cl^–^]_i_ transients. This is consistent with the idea that reducing KCC2 activity degrades Cl^–^ homeostasis and the ability to restore [Cl^–^]_i_ and the proper polarity of GABA_A_ signaling after synaptic activity. KCC2 antagonists have a potential anti-ictal effect in their reduction of [K^+^]_0_ transients. We did not find evidence for an anti-ictal effect, but our slice cultures were perfused via the bath rather than via the vasculature, so it is possible that bulk ACSF flow reduced this potential anti-ictal effect on KCC2-dependent [K^+^]_0_ accumulation (Viitanen et al., 2010).

There is substantial interest in overexpression and enhancement of KCC2 transport activity as a novel therapeutic strategy to improve synaptic GABAergic inhibition in neurologic disorders (Kahle et al., 2008; Gagnon et al., 2013; Moore et al., 2018; Magloire et al., 2019). High-throughput screening identified a new high-affinity compound CLP257 (EC_50_ = 616 nm) and its carbamate prodrug derivative CLP290 that selectively activates KCC2 over other KCC family members, NKCC1 and GABA_A_ receptors (Gagnon et al., 2013). CLP257 restored impaired Cl^–^ transport in adult spinal cord slices with impaired KCC2 function, restored Cl^–^ extrusion and renormalized stimulus-evoked responses in adult neurons in an experimental model of neuropathic pain. However, the pharmacodynamic profile of CLP257, including KCC2 specificity and mode of action, has not been determined. High concentrations of CLP257 (30–50 μm) was reported to potentiate GABA_A_ receptors (Cardarelli et al., 2017). Even higher concentrations of CLP257 (100 μm) increased the duration of ILDs without affecting their frequency (Hamidi and Avoli, 2015).

We investigated whether enhancing KCC2 transport activity with a low concentration of CLP257 affects baseline [Cl^–^]_i_ and [Cl^–^]_i_ extrusion rates in injured neurons during ictal-like events, and the downstream effects on frequency and duration of recurrent ILDs in the organotypic hippocampal slice *in vitro* model of epileptogenesis (Fig. 8). We found that low concentrations of CLP257 (1 μm) improved [Cl^–^]_i_ homeostasis, increased postictal [Cl^–^]_i_ extrusion rates, and modestly reduced ictal duration without effecting ictal frequency. Our data suggest that the modest anticonvulsant actions of low dose CLP257 are likely partially mediated by enhanced KCC2 transport activity that more efficiently restores neuronal baseline Cl^–^ during termination of ictal-like events. In addition, our data validate CLP257 as a promising target of investigation for antiepileptic therapy and highlight the ongoing need to develop more specific activators of KCC2 cotransport.

## Synthesis

Reviewing Editor: Alfonso Represa, INSERM Decisions are customarily a result of the Reviewing Editor and the peer reviewers coming together and discussing their recommendations until a consensus is reached. When revisions are invited, a fact-based synthesis statement explaining their decision and outlining what is needed to prepare a revision will be listed below. The following reviewer(s) agreed to reveal their identity: NONE.

This manuscript explores the consequences of pharmacological manipulations of cation/chloride cotransporters on spontaneous pathological activities recorded from mouse hippocampal organotypic slice cultures. The authors combine field recordings with live imaging of the chloride probe clomeleon Overall the paper is well written, data are convincing and the topic is important due to the involvement of KCC2 in epilepsy. There are however number of concerns that need careful revision:

1) The title of the manuscript is misleading as it refers to ߢseizuresߣ, as if experiments were conducted in vivo. In the rest of the manuscript (except the figures), the authors more carefully name epileptiform activities recorded from organotypic cultures ߢictal like dischargesߣ, which is appropriate. The title should not infer that the in vitro data apply to in vivo seizures, unless demonstrated experimentally. We suggest to replace ߢseizuresߣ with ߢictal-like dischargesߣ throughout the manuscript.

2) Authors should specify the « in vitro » model used in the study in the abstract.

3) Another difficulty with this manuscript concerns the metrics, which vary depending on the pharmacological treatments, making it very hard to the reader to compare the effects of the various treatments. Thus, the authors sometimes use the mean frequency and power of ictal like discharges as a metric for the effect of a drug, while in other cases the duration of ILDs are provided. Similarly, the mean ILD frequency is sometimes plotted over time or only averaged, depending on the treatment. The authors should put some effort representing their data in a consistent manner throughout their manuscript in order to help comparison of the effects of their various treatments.

4) Related to this issue, it is difficulty to really figure out the effect of each drug or drug combination. For instance, the authors refer page 13 to the ߢepileptogenic effect of VU04663271ߣ though authors just showed in figure 2D that VU04663271 actually reduces ILDs from 4 down to about 1 per 10 minutes. The authors should clarify how they end up with 1.83/10 min as stated in page 11. Authors must clarify how mean values were derived throughout the ms.

5) Most of the drugs used in this study are dissolved in DMSO (notably CLP257 and VU drugs). DMSO has been shown to increase neuronal activity through mechanisms that remain poorly understood. Therefore, the appropriate control for these compounds is not just saline, but saline supplemented with equimolar concentrations of DMSO. Authors should at least test the effect of DMSO at concentrations used for drug application on network activity in their model.

6) Figure 1.

Where peak intracellular Cl- concentration during seizures and SE similar at DIV6-8 vs DIV27-28?

Fig 1D: Was seizure frequency affected by the occurrence of SE in slices?

Fig 1E-F: TTX experiments. “Although TTX changed the baseline [Cl-] i , it did not change the kinetics of postictal [Cl-] i recovery (Fig. 1E, F)”. I do not really understand how authors compare the Cl- decay since TTX blocks the seizures. The decay after the last seizure blocked by TTX is certainly involving very different mechanisms. I suggest to remove this part.

Fig 1G: Neuronal Cl- concentration drop at DIV7. Is it correlated with Cl- cotransporters expression (at least KCC2)?

7) Figure 2.

Fig 2E-F. As in figure 1 I do not find decay measurements after a seizure event comparable to TTX induced shutdown.

Did KCC2 blockade result in transformation of ictal like events to SE in DIV6-8 slices?

8) Figure 3.

Fig 3A: What are the effects of GABA-A receptors blockade on neuronal Cl-?

Fig 3C: may the authors add an expanded trace of seizures before BUM applications?

Usefulness of blocking KCC2 by Bumetanide and then checking that complementary blockade by VU04663271 has no extra effect is limited. Why not testing, with a control of neuronal Cl-, low doses of Bumetanide, providing a specific blockade of NKCC1, and then block KCC2? And vice versa?

9) Figure 4.

Fig 4A-C: May the authors provide expanded traces of seizure like events before and after Furosemide applications? What is really happening with low doses of Furosemide? Longer seizures or exclusive interictal spikes?

Page 16:” These data also suggest that the anti-seizure effects of high concentrations of furosemide are due to interactions with an unidentified Cl - transporter, exchanger, or channel”. This is expected since Furosemide also blocks NKCC1!

Why not comparing the decay of neuronal Cl- concentration during the last seizure blocked by TTX vs blocked by high concentrations of Furosemide?

10) Figure 5.

There is an apparent contradiction with Figure 4D showing that furosemide alone reduces ILD frequency down to zero, and in all figure is a bit confusing. If the authors wish to compare the effect of furosemide in the presence vs. absence of KCC2 or NKCC1 antagonist, they should provide statistics.

Why not displaying Cl- concentration data? Especially after low doses of Bumetanide applications.

Bumetanide effects usually take time since it reduces Cl- loading but require Cl- extrusion to process. May the authors study the effects of Bumetanide on longer time periods? Are seizures like events totally blocked 60 min after Bumetanide?

Page 16: “In contract, furosemide (1 mM) strongly enhanced...” I think the authors mean contrast.

If the authors wish to investigate the action of furosemide beyond CCC (which is already well documented in the literature (PMID: 9138676 should be appropriately quoted here), they should perform cellular electrophysiology experiments, as network activity and intraneuronal chloride are not appropriate metrics to conclude on this question.

11) Figure 6.

What where the effects of 100 microM CLP257 on neuronal Cl- concentration at rest, at peak during seizure-like events and during decays?

Lactate and LDH measurements are out of the scope of this study. I suggest withdrawing them.

12) Page 4, para. 2. There is a current controversy as to whether KCC2 may or may not be a selective KCC2 enhancer, as it seems to also act as a GABAAR allosteric modulator (Cardarelli et al Nat Med 2017). The authors should at least mention this and be more careful and name this compound ߢputative KCC2 enhancerߣ rather than ߢselective KCC2 activatorߣ, which it is clearly not. To note that authors tend to restrict the literature citation to their own contributions letting aside other important contributions.

13) Discussion, P20: “When effects on seizure activity were removed, KCC2 modulators had no effect on baseline [Cl- ] i “ It is not really demonstrated in this study since the only evaluation of CCC modulation, out of epileptic activity, was performed by blocking both Cl- load and exclusion by Furosemide.

14) It would be most helpful if the authors put some effort trying to synthesize their observations in their discussion and provide a visual abstract of their major conclusions.

15) The authors should provide a more balanced references citation, taking into account the contribution of other than their own group to a rather abundant and rich literature.
